# Development of new analytical methods for the determination of caffeine content in aqueous solution of green coffee beans

**DOI:** 10.1186/s13065-017-0356-3

**Published:** 2017-12-05

**Authors:** Blen Weldegebreal, Mesfin Redi-Abshiro, Bhagwan Singh Chandravanshi

**Affiliations:** Department of Chemistry, College of Natural Sciences, Addis Ababa University, P.O. Box 1176, Addis Ababa, Ethiopia

**Keywords:** Green coffee beans, Caffeine, FT-IR-ATR, NIR, Fluorescence spectroscopy

## Abstract

**Background:**

This study was conducted to develop fast and cost effective methods for the determination of caffeine in green coffee beans. In the present work direct determination of caffeine in aqueous solution of green coffee bean was performed using FT-IR-ATR and fluorescence spectrophotometry. Caffeine was also directly determined in dimethylformamide solution using NIR spectroscopy with univariate calibration technique.

**Results:**

The percentage of caffeine for the same sample of green coffee beans was determined using the three newly developed methods. The caffeine content of the green coffee beans was found to be 1.52 ± 0.09 (% w/w) using FT-IR-ATR, 1.50 ± 0.14 (% w/w) using NIR and 1.50 ± 0.05 (% w/w) using fluorescence spectroscopy. The means of the three methods were compared by applying one way analysis of variance and at p = 0.05 significance level the means were not significantly different. The percentage of caffeine in the same sample of green coffee bean was also determined by using the literature reported UV/Vis spectrophotometric method for comparison and found to be 1.40 ± 0.02 (% w/w).

**Conclusion:**

New simple, rapid and inexpensive methods were developed for direct determination of caffeine content in aqueous solution of green coffee beans using FT-IR-ATR and fluorescence spectrophotometries. NIR spectrophotometry can also be used as alternative choice of caffeine determination using reduced amount of organic solvent (dimethylformamide) and univariate calibration technique. These analytical methods may therefore, be recommended for the rapid, simple, safe and cost effective determination of caffeine in green coffee beans.

## Background

The name coffee is derived from the name of the province Keffa where shepherds from Abyssinia/Ethiopia discovered the coffee plant in the 6th century. Since then coffee has become one of the most widely consumed beverages throughout the world due to its pleasant taste, aroma, stimulant effect and health benefits [1]. Coffee comprises more than 90 different numbers of species. However, only *Coffea arabica*, *robusta*, and *liberica* are of commercial importance. *Coffea arabica* accounts for approximately 75% while *robusta* accounts for about 25% and *liberica* (< 1%) of the world’s production, other species are of not much commercial value [2].

Drinking coffee, called “Bunna” in Amharic is an important element of cultural beverage in Ethiopia. Coffee is the second important raw material within the international trade, the most important foreign exchange supplier for many agricultural oriented countries, an attractive source for tax yield, and the most popular drink. Due to the economic importance of coffee there is an increasing demand for proper quality control for certification of contents and substandard products. Therefore, sensitive and accurate analytical methods for both qualitative and quantitative determinations and characterization of chemical substances in coffee are required.

Coffee has many volatile and non-volatile components. In addition to caffeine, coffee contains substantial amounts of bioactive components which are a family of conjugated hydroxycinnamates, collectively referred to as chlorogenic acids, diterpenes and trigonelline [3]. The chemical composition of green coffee mainly depends on the variety of the coffee, although slight variations are possible due to agro-climatic conditions, agricultural practices, and processing and storage.

Caffeine (1,3,7-trimethylxanthine) is the active alkaloid component which is a naturally occurring substance found in the leaves, seeds or fruits of over 63 plants species worldwide. The world’s primary source of caffeine is the coffee bean which is actually the seed of the coffee plant [4]. Green coffee beans of *Coffea arabica* contains between 0.7 and 1.6% caffeine and of *Coffea robusta* between 1.5 and 4.0% caffeine [5]. Caffeine is provided through a number of different sources, most commonly through coffee, tea and soft drinks. It was consumed daily in coffee, tea, cocoa, chocolate, some soft drinks, energy drinks and some drugs.

Caffeine acts as central nervous system stimulant that increases alertness, reduced sleep, improves short term memory and increases the effectiveness of certain drugs [6]. Caffeine has been enjoyed by humans for many years through consumption of foods and beverages containing caffeine including of coffee beverage. Hence, it is important to develop simple analytical methods in order to characterize and identify the amount of caffeine in coffee beans.

Many analytical methods have been developed for the determination of caffeine in coffee beans and products containing caffeine including electroanalytical [7, 8]; chromatographic [4, 9, 10] techniques including gas chromatography [11–13], high performance liquid chromatography (HPLC) [4, 10, 14, 15], liquid chromatography-particle beam/electron ionization mass spectrophotometry [16], liquid chromatography-tandem mass spectrometry [17], and spectroscopic techniques [1, 4, 18–22] including nuclear magnetic resonance spectroscopy [23], near infrared spectroscopy [24, 25], near infra-red reflectance spectroscopy [26], and UV–Vis spectroscopy [1, 20, 27, 28], and fluorescence polarization immunoassays [29]. HPLC is the method of choice by many researchers in determining the caffeine contents of beverages, tea leaves and coffee beans. However, HPLC is a high-priced, resource consuming and technically demanding even that is not typically found in most universities especially in developing countries such as Ethiopia.

As different literatures indicated spectrophotometric determination of caffeine is also reported as preferred method of determination such as UV–Vis spectrophotometry because of its relatively low cost, rapidity, high accuracy and reproducibility. But UV–Vis spectrophotometric method cannot be used directly for determination of caffeine in coffee beans extracted with water owing to the matrix effect of UV–Vis absorbing substances in the sample matrix [9]. In aqueous solution of coffee beans it was observed that there is spectral interference from caffeine and chlorogenic acid in the wavelength regions of 200–500 nm. Yet this method requires the extraction of caffeine from the aqueous solution of coffee beans using dichloromethane for the spectroscopic determination. This is necessary since the caffeine spectrum is overlapped with other compounds found in coffee. Hence, the use of dichloromethane limits the wider application of UV–Vis method.

Therefore, this research was aimed to investigate the possibility of spectroscopic methods for the determination of caffeine in aqueous solution of green coffee beans by developing simple, fast and cost effective procedures. This is because the amount of caffeine from coffee bean is taken by human beings through drinking of coffee beverage prepared in hot water as the extracting medium. Hence, it is always desirable to develop a method which is similar with the actual conditions to assess the actual intake of caffeine through coffee.

Hence this study was conducted to develop fast and cost effective methods for the determination of caffeine in green coffee beans. In the present work direct determination of caffeine in aqueous solution of green coffee bean was performed by using FT-IR-ATR and fluorescence spectrophotometry. Caffeine was also directly determined in dimethylformamide solution using NIR spectroscopy with univariate calibration technique.

## Experimental

### Apparatus and instruments

Electronic balance (ARA520, OHAUS CORP., China) was used to measure the mass of standard and green coffee bean samples. Magnetic stirrer with a hot plate (Model 04803-02, Cole Parmer, 230 V, 50 Hz, and 2 Amp, USA) was utilized to dissolve the standard and the green coffee bean samples. Blending device (Electric motor grinder) (GEEPAS CR., Main land, China) was used for grinding green coffee bean samples. Hitachi spectrofluorimeter (Flouromax-4, spectrofluorimeter, USA) with 1 cm quartz cuvette were used to record the excitation and emission spectrum of the solution. Electronic absorption of the solution was recorded using Perkin Elmer instruments. For the UV/Vis and NIR measurements 1 cm quartz cuvette and a double beam UV–Vis-NIR spectrometer (Perkin Elmer Lambda 950, Llantrisant, CF728YW, UK) with wavelength regions 170–3200 nm were used. For the mid IR measurement a sample holder of zinc selenide crystal and Fourier transform (Perkin Elmer, spectrum 65 spectrophotometer, USA) with wave number range 4000-400 cm^−1^ were used.

### Chemicals and samples

Standard caffeine (Fishel company, Germany), *N,N*-dimethylformamide (Riedel-de Haen, 99%), acetone (Sigma-Aldrich, 99%) and dichloromethane (Sigma-Aldrich, 99%) were used. The coffee sample was collected from local market without considering its variety. Distilled deionized water was used in all experimental work. The distilled deionized water used was prepared in our laboratory by the glass distiller followed by purification by passing through an ion-exchanger.

### Standard caffeine solutions for FT-IR spectrometry

A 9942 mg/L stock standard solution of caffeine was prepared by dissolving 0.5 g of standard caffeine with 40 g of distilled water and diluted to final weight of 50.29 g in 100 mL volumetric flask. Working standards were prepared by weighing 1.00, 2.01, 3.017, 4.023, 5.03 and 6.035 g, respectively, aliquots of the stock standard solution were transferred into separate volumetric flasks (25 mL). All the aliquots were diluted to 10 g of final weight of the solution with distilled water to produce concentrations of 1000, 2000, 3000, 4000, 5000 and 6000 mg/L standard solution, respectively, for the FT-IR-ATR calibration measurement. The maximum peak of absorption of the aqueous solution of standard caffeine was obtained by scanning the standard solution from 4000-400 cm^−1^ and the spectrum over the wavenumber range (2825–2982) cm^−1^ with a good absorption spectrum of standard caffeine was selected for quantitative determination.

### Standard caffeine solutions for NIR spectroscopy

A 9847 mg/L stock solution of standard caffeine was prepared by dissolving 0.47 g of standard caffeine in 40 g *N*,*N*-dimethylformamide (DMF) in 100 mL beaker and diluted to 47.73 g of final weight of the solution in 100 mL volumetric flask. Working standards were prepared by weighing 1.01, 2.03, 3.04, 4.06 and 5.07 g aliquots of the standard stock solution into separate 25 mL volumetric flask. Each aliquot was diluted to 10 g of final weight of the solution with DMF to produce concentrations of 1000, 2000, 3000, 4000 and 5000 mg/L, respectively. The absorbance of the solution was measured in the range of 1200–2110 nm against the corresponding reagent blank (DMF).

### Standard caffeine solutions for fluorescence spectroscopy

The stock solution 970 mg/L of standard caffeine was prepared by dissolving 0.97 g of standard caffeine in 300 mL distilled water and diluted to 1 L in a volumetric flask. Another less concentrated solution (19.4 mg/L) was prepared from the stock solution by applying weight to weight dilution. Working standards were prepared by weighing 0.76, 1.53, 3.10, 5.89, and 11.34 g, respectively, added aliquots of the standard solution into separate 50 mL volumetric flask and diluting to 25 g final weight of the solution with distilled water to produce concentrations of 0.594, 1.19, 2.40, 4.58, and 8.80 mg/L, respectively. The excitation wavelength at 272 nm and the λ_max_ of emission was determined by scanning the standard solution 250–500 nm. The spectrum was best at 385 nm, which was far from the Rayleigh and Raman scattering. For quantitative determination the excitation property was used. The emission wavelength was set at λ_max_ = 385 nm and scanned the solutions over the range 240–360 nm to obtain the maximum excitation intensity.

### Sample preparation

Green coffee beans were ground and screened through 250 μm sieve to get a uniform texture. Then accurately weighed amount of sieved coffee was dissolved in distilled water for fluorescence and FT-IR analysis and in DMF for NIR determination. The solution was stirred using magnetic stirrer and heated gently to remove caffeine easily from the solution. The time of extraction was 60 min. The solution was filtered through Whatman filter paper to get clear solutions. Finally the filtrate of green coffee beans was directly used for qualitative and quantitative analysis by using spectrophotometric techniques (UV–Vis-NIR, fluorescence and FT-IR-ATR).

### Statistical analysis

For the all methods triplicate measurements of sample were performed. The results were expressed as mean ± standard deviation for all replicate measurements. The data obtained were statistically analyzed by using origin statistical software (version 6.0). The data were also subjected to one way analysis of variance (ANOVA) using origin soft ware (version 6.0) to test the significance differences in the mean values of caffeine obtained by the three methods (NIR, FT-IT-ATR, and fluorimetry).

## Results and discussion

### Determination of caffeine content by FT-IR-ATR method

**Fig. 1:**
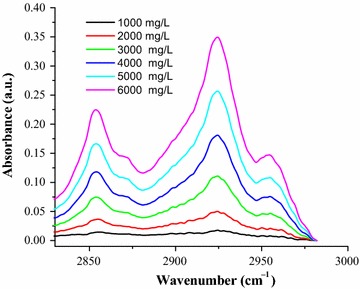
FT-IR-ATR absorption spectra of standard caffeine in water

To determine the percentage of caffeine in aqueous solution of green coffee beans six working solutions of standard caffeine in the range of (1000–6000 mg/L) were prepared and the absorption spectra of the standard solutions were measured over a wavenumber range (2825–2982) cm^−1^ (Fig. 1).

**Fig. 2:**
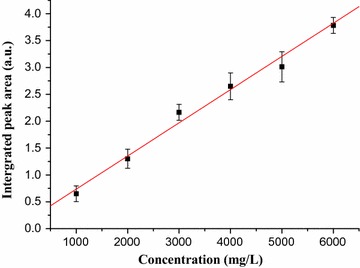
Graph of concentration versus integrated peak area for standard caffeine in water

The obtained spectrum was treated with baseline correction and separately integrated over the range (2982–2882) and (2880–2825 cm^−1^). The peak area was obtained from the integrated FT-IR-ATR spectrum by adding the peak areas integrated separately. Then the integrated peak area versus concentration graph (Fig. 2) was constructed.

**Fig. 3:**
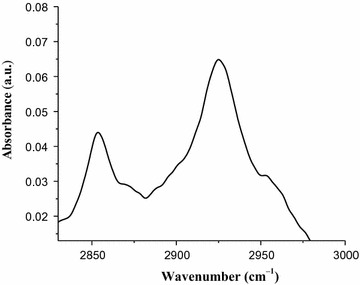
FT-IR-ATR absorption spectrum of green coffee beans dissolved in water

The calibration curve obtained for FT-IR-ATR determination of caffeine had correlation coefficient (R = 0.993) and the calibration curve was linear over the range (1000–6000) mg/L of standard caffeine with equation (y = 0.13045 + 0.000608x, where, y indicates the sum of integrated peak area and x indicates concentration in mg/L). The amount of caffeine in aqueous solution of green coffee bean (mg/L) was determined using the calibration curve. Finally, the percentage of caffeine (Table 1) was calculated by taking the mass of caffeine calculated from the calibration curve (Fig. 3).

**Table 1:** The mean percentage of caffeine obtained by the three methods

Methods	Mass of coffee (g)	Mass of solution (g)^a^	Mass of caffeine (g)	Caffeine in coffee (% w/w)	Mean ± SD (% w/w)
FT-IR-ATR	2.05 2.00 2.00	10.00 10.05 10.00	0.0334 0.0293 0.0294	1.629 1.465 1.470	1.520 ± 0.093
NIR	2.05 2.00 2.00	10.00 9.980 10.00	0.0343 0.0289 0.0281	1.680 1.440 1.410	1.500 ± 0.14
Fluorescence	0.5 0.5 0.5	65 75 70	0.0005682 0.0005255 0.0005624	1.434 1.530 1.529	1.497 ± 0.05
UV-Vis (for comparison)	0.33 0.33 0.33	Extracting volume 100 mL	0.00453 0.00465 0.00466	1.373 1.409 1.410	1.397 ± 0.02

^a^The mass of the solution was measured to avoid any changes in the concentration which may results from the changes in the volume of the solution

The standard caffeine dissolved in water and the filtrate of aqueous coffee solution showed similar FT-IR absorption spectra over the wavenumber range (2825–2982) cm^−1^ which showed a maximum absorption at around 2855 and 2924 cm^−1^. The two spectra were exactly similar to each other both in peaks and shapes. The similarity in peak and shape of the two spectra show there is no overlap band from other components of coffee in these regions, and this shows the specificity of the method. The use ATR accessories in conjunction with FT-IR spectrometers provides for the non-destructive measurement of sample and the ATR accessory also allows for easy and reproducible as well as fast analysis of liquid samples with just a few drops required.

FT-IR-ATR determination of caffeine in aqueous solution of green coffee beans was characterized with two sharp peaks at around 2855 and 2924 cm^−1^; these bands are correlated with the symmetrical and asymmetrical stretching of C–H bonds of methyl (–CH_3_) group in the caffeine molecule and the absorption region over the wavenumber range of 2982–2825 cm^−1^ was successfully used for quantitative determination of caffeine in green coffee beans. Hence this stretching vibration may play an important role in the qualitative and quantitative analysis of caffeine in aqueous solution of coffee beans.

There are also other FT-IR literature data on coffee obtained by transmission and reflectance techniques with similar spectrum in which the two sharp bands that can be viewed in the 3000–2800 cm^−1^ have been reported qualitatively for both *C*. *arabica* and *C*. *robusta* coffee samples [30]. Studies of FT-IR analysis of caffeine on soft drinks have also reported two sharp peaks at 2882 and 2829 cm^−1^, the peak region being successfully used to for quantitative analysis of caffeine [19].

### Determination of caffeine content by NIR spectroscopy method

**Fig. 4:**
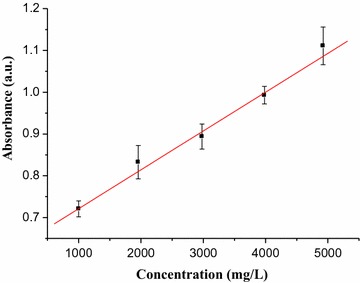
Absorbance versus concentration graph of standard caffeine in DMF

To determine the percentage of caffeine in DMF solution of green coffee beans five working solutions of standard caffeine in the range of (1000–5000 mg/L) were prepared and the absorbance versus concentration graph (Fig. 4) was constructed. The calibration curve obtained for NIR determination of caffeine had correlation coefficient (R = 0.994) and the standard calibration curve was linear over the range (1000–5000) mg/L of standard caffeine in DMF with equation (y = 0.62786 + 9.51 × 10^−5^x, where y indicates maximum absorbance and x indicates concentration in mg/L). The quantitative amount of caffeine in DMF solution of green coffee bean (mg/L) was determined using the calibration curve. Finally, the percentage of caffeine (Table 1) was calculated by taking the mass of caffeine calculated from the linear calibration curve.

NIR spectrophotometric method cannot be used directly for the determination of caffeine in aqueous solution of green coffee beans. In the NIR region water absorbs strongly, the free spectral range is not wide and on the free spectral range available the absorption of aqueous solution of caffeine is not significant. Therefore, it is necessarily to use other solvents which are available for the NIR determination of caffeine in coffee beans. For this method, DMF was selected as a solvent which is less carcinogenic than chlorinated solvents, its ability to dissolve caffeine very well and having free spectral range on the studied region.

**Fig. 5:**
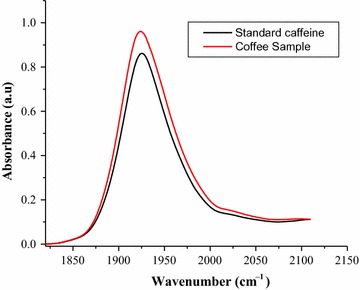
NIR spectrum of standard caffeine and coffee dissolved in DMF

From the spectrum shown in Fig. 5 the NIR spectra of standard caffeine and the filtrate of coffee bean solution in DMF have strong similarity. The two spectra are qualitatively similar. Hence, the region over the range (2110–1820 nm) was used for quantitative determination of caffeine in green coffee beans.

A method of caffeine determination in coffee beans using univariate calibration technique was developed in the present study which can overcome the difficulty of NIR region for direct determination of caffeine in green coffee beans. Regarding caffeine content determination a fast, simple and cost effective procedure was developed using NIR spectrophotometry in green coffee bean samples with reduced amount of organic solvent used. The sensitivity of spectrometric measurements relies on band intensities, even the spectra obtained for the NIR measurement of caffeine in DMF was very intense band relative with other less intense bands in which spectral information is repeated throughout the successive overtones and combination regions.

### Determination of caffeine content by fluorescence method

**Fig. 6:**
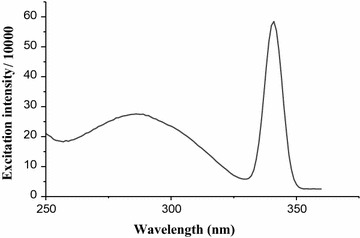
Fluorescence excitation spectrum of standard caffeine in water

The standard caffeine dissolved in water and the aqueous solutions of green coffee beans showed an emission and excitation spectrum. However, there is difficulty for quantification of caffeine in aqueous solution of coffee beans using the emission property due to strong overlapping. Therefore, to overcome this difficulty it is necessary to quantify the amount of caffeine using the excitation intensity. Hence, fluorescent compounds can be identified or quantified on the basis of their excitation or emission properties. The fluorescence excitation intensity versus wavelength spectrum of standard caffeine is shown in Fig. 6.

**Fig. 7:**
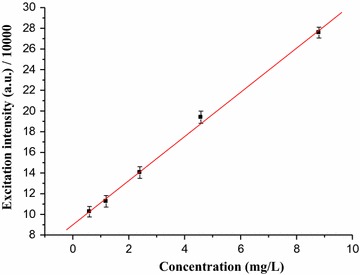
Graph of maximum excitation intensity *vs* concentration of standard caffeine

**Fig. 8:**
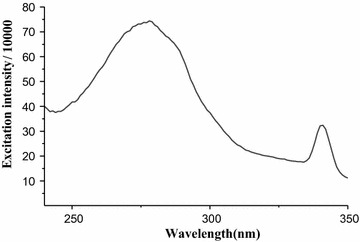
Fluorescence excitation spectrum of coffee dissolved in water

To determine the percentage of caffeine in aqueous solution of green coffee beans five working solutions of standard caffeine in the range of (0.59–8.8 mg/L) were prepared and the absorbance versus concentration graph (Fig. 7) was constructed. From the calibration curve correlation coefficient was (R = 0.998) and the calibration curve was linear over the range with equation (y = 4.15867 × 10^9^ x + 8.974 × 10^4^, where y indicates maximum excitation intensity and x indicates concentration). The quantitative amount of caffeine in aqueous solution of green coffee bean (mg/L) was then determined using the calibration curve. Finally, the percentage of caffeine (Table 1) was calculated by taking the mass of caffeine calculated from the linear calibration curve. The fluorescence excitation spectrum of coffee beans dissolved in water is shown in Fig. 8. One can clearly see that the maximum absorbance–wavelength of standard caffeine in water (Fig. 6) and coffee dissolved in water (Fig. 8) are almost the same. The differences in the peak area are due to differences in the concentration of caffeine.

Using the proposed method the percentage of caffeine in aqueous solution of green coffee beans was determined employing fluorescence spectrometry. It was determined from the excitation intensity of caffeine setting the emission wavelength on 385 nm and scanning over the range (240–360 nm) to collect the maximum excitation intensity.

The present methods are simple, rapid and cost effective in which water is used for the whole experimental parts. The percentage of caffeine in aqueous solution of green coffee beans was directly investigated using FT-IR-ATR and fluorescence spectrophotometries. The percentage of caffeine in dimethylformamide solution of green coffee beans was also directly investigated using NIR spectrophotometry with reduced amount of organic solvent used. The sample was collected from a local market in which the origin of the coffee sample is not known. The target of the present work was not to determine and compare the percentage of caffeine in coffee beans cultivated in different areas rather it was to validate the developed fast, accurate and cost effective methods for caffeine determination.

Table 1 shows that the results obtained are comparable with the highest caffeine content of *C*. *arabica* coffee samples as reported by [1] for different Ethiopian *C*. *arabica* coffee samples grown in Wembera, Goncha, Zegie and Burie determined by UV–Vis spectrophotometry using dichloromethane for extraction to be 1.53 ± 0.003, 1.41 ± 0.04, 1.29 ± 0.033 and 0.97 ± 0.049 (% w/w), respectively. Another study using HPLC method also showed caffeine content variability as reported by [9] ranging from 0.6 to 1.21, 0.7 to 1.82 and 0.9 to 1.62% among 9, 21 and 38 *C*. *arabica* genotypes, respectively. Therefore, these values are in reasonable degree of agreement with the value of the present work. A recent study using HPLC method also showed caffeine content variability as reported by [10] ranging from 0.87 to 1.38% of caffeine among 100 coffee *C*. *arabica* samples from different regions of Ethiopia.

Studies have indicated that the chemical composition of green coffee beans mainly depends on the variety of the coffee, although slight variations are possible due to agro-climatic conditions, agricultural practices (processing and storage), its species, origin and weather of the plantation [1, 10, 31]. Hence the variation of caffeine content of coffee samples may be due to the difference come from geographical origins.

### Comparison of results obtained by three newly developed methods for caffeine determination

In the present study, three different methods were developed for the quantitative determination of caffeine in green coffee beans by using water and DMF as a solvent employing the same procedure for all methods. Hence, all the results were comparable with the percentage of caffeine in *C*. *arabica* green coffee beans determined by using other methods such as UV/Vis spectrophotometry and HPLC method. The analytical parameters such as correlation coefficient (R), linear range, limit of detection (LOD), limit of quantification (LOQ) and relative standard deviation (RSD) of each method are given in Table 2.

**Table 2:** The analytical parameters for the three developed methods

Methods	Liner range	R	LOD	LOQ	RSD (%)
FT-IR-ATR	(1–6) g/L	0.993	0.15 g/L	0.5 g/L	5.9
NIR	(1–5) g/L	0.994	0.3 g/L	1 g/L	9.3*
Fluorescence	(5.95 × 10^−4^–87.3 × 10^−4^) g/L	0.998	1.75 × 10^−4^ g/L	5.82 × 10^−4^ g/L	3.7

*The relatively higher RSD may be attributed to the high background absorption of solvent water which results in higher noise level

The data were also subjected to one way analysis of variance (ANOVA) using origin soft ware (version 6.0). The ANOVA results indicated that at 5% significance level, the means for the three methods are not significantly different.

### Comparison of results obtained by the present developed methods with UV/Vis spectrophotometry

To validate the newly developed methods it is necessary to compare the results using standard method or with other accepted methods. The present methods developed for caffeine determination were compared with the results obtained by using literature reported UV/Vis spectrophotometric methods. The UV/Vis spectrophotometric methods have been reported by many researchers as preferred method of caffeine determination because of its relatively low cost, rapidity, high accuracy and reproducibility.

Belay et al. [20] reported that UV/Vis spectrophotometer cannot be used directly for determination of caffeine in aqueous solution of coffee due to sample matrix effect. To overcome this difficulty the coffee samples was first dissolved in water and extracted with dichloromethane based on the procedure developed by Belay et al. [20]. After extraction, the absorbance of the solution was measured using UV/Vis spectrophotometer and the maximum absorbance was obtained at 275 nm. The mean percentage of caffeine determined from UV/Vis analysis of green coffee beans extracted using dichloromethane (extracting volume 100 mL) is given in Table 1.

The results obtained using the three newly developed methods are comparable with the results obtained using UV/Vis spectrophotometry [1] and HPLC [9, 10] from literature (Table 3). This was further confirmed by applying t test to compare the means of the three newly developed methods with the mean of caffeine obtained by using UV/Vis spectrophotometry for the same coffee sample. The results indicated that at 95% confidence level the means are not significantly different.

**Table 3:** Comparison of the means of each of the three newly developed methods with the mean obtained by UV/Vis spectrophotometer using t test at 95% confidence level

Methods	Mean ± SD (%)	Degree of freedom	t_calculated_	t_critical_	Remark
FT-IR-ATR	1.52 ± 0.093	4	2.05	2.132	No significantly different
NIR	1.50 ± 0.14	4	1.26	2.132	No significantly different
Fluorescence	1.50 ± 0.05	4	1.97	2.132	No significantly different

## Conclusion

Two simple, rapid and inexpensive methods were developed for direct determination of caffeine content in aqueous solution of green coffee beans using FT-IR-ATR and fluorescence spectrophotometries. Water was used for the whole process which is the cheapest solvent found everywhere, environmentally friendly and can help to perform experiments without suffering from the toxic nature of different organic solvents. NIR spectrophotometry can also be used as alternative choice of method for caffeine determination using reduced amount of organic solvent (dimethylformamide) and univariate calibration technique. Therefore, a quantitative determination of caffeine in green coffee beans become feasible by employing the present proposed spectroscopic methods with simple, short time of analysis and inexpensive procedure. In addition the methods have been tested for roasted coffee beans and are applicable with the same procedure. These analytical methods may therefore, be recommended for the rapid, simple, safe and cost effective determination of caffeine in coffee beans.
